# *Haemophilus ducreyi* DNA is detectable on the skin of asymptomatic children, flies and fomites in villages of Papua New Guinea

**DOI:** 10.1371/journal.pntd.0004958

**Published:** 2017-05-10

**Authors:** Wendy Houinei, Charmie Godornes, August Kapa, Sascha Knauf, Eric Q. Mooring, Camila González-Beiras, Ronald Watup, Raymond Paru, Paul Advent, Sivauk Bieb, Sergi Sanz, Quique Bassat, Stanley M. Spinola, Sheila A. Lukehart, Oriol Mitjà

**Affiliations:** 1 Disease Control Branch, National Department of Health, Port Moresby, Papua New Guinea; 2 Department of Medicine, University of Washington, Seattle, Washington, United States of America; 3 Lihir Medical Centre– International SOS, Newcrest Mining, Lihir Island, Papua New Guinea; 4 German Primate Center, Leibniz-Institute for Primate Research, Pathology Unit, Working Group Neglected Tropical Diseases, Göttingen, Germany; 5 Department of Epidemiology, Harvard T.H. Chan School of Public Health, Boston, Massachusetts, United States of America; 6 Global Public Health PhD Programme, Nova University of Lisbon, Lisbon, Portugal; 7 Barcelona Institute for Global Health (ISGlobal), Hospital Clinic-University of Barcelona, Barcelona, Spain; 8 Centro de Investigação em Saúde de Manhiça (CISM), Maputo, Mozambique; 9 Institució Catalana de Recerca i Estudis Avançats (ICREA), Barcelona, Spain; 10 Departments of Microbiology and Immunology, Medicine, and Pathology and Laboratory Medicine Indiana University School of Medicine, Indianapolis, Indiana, United States of America; 11 Department of Global Health, University of Washington, Seattle, Washington, United States of America; 12 Division of Public Health, School of Medicine and Health Sciences, University of Papua New Guinea, Port Moresby, Papua New Guinea; Task Force for Child Survival and Developmentorce for Global Health, UNITED STATES

## Abstract

**Background:**

*Haemophilus ducreyi* and *Treponema pallidum* subsp. *pertenue* are major causes of leg ulcers in children in Africa and the Pacific Region. We investigated the presence of DNA (PCR positivity) from these bacteria on asymptomatic people, flies, and household linens in an endemic setting.

**Methodology/Principal findings:**

We performed a cross-sectional study in rural villages of Lihir Island, Papua New Guinea during a yaws elimination campaign. Participants were asymptomatic subjects recruited from households with cases of leg ulcers, and from households without cases of leg ulcers. We rubbed swabs on the intact skin of the leg of asymptomatic individuals, and collected flies and swabs of environmental surfaces. All specimens were tested by PCR for *H*. *ducreyi* and *T*. *p*. *pertenue* DNA. Of 78 asymptomatic participants that had an adequate specimen for DNA detection, *H*. *ducreyi-*PCR positivity was identified in 16 (21%) and *T*. *p*. *pertenue*-PCR positivity in 1 (1%). In subgroup analyses, *H*. *ducreyi-*PCR positivity did not differ in participants exposed or not exposed to a case of *H*. *ducreyi* ulcer in the household (24% vs 18%; p = 0.76). Of 17 cultures obtained from asymptomatic participants, 2 (12%) yielded a definitive diagnosis of *H*. *ducreyi*, proving skin colonization. Of 10 flies tested, 9 (90%) had *H*. *ducreyi* DNA and 5 (50%) had *T*. *p*. *pertenue* DNA. Of 6 bed sheets sampled, 2 (33%) had *H*. *ducreyi* DNA and 1 (17%) had *T*. *p*. *pertenue* DNA.

**Conclusions/Significance:**

This is the first time that *H*. *ducreyi* DNA and colonization has been demonstrated on the skin of asymptomatic children and that *H*. *ducreyi* DNA and *T*. *p*. *pertenue* DNA has been identified in flies and on fomites. The ubiquity of *H*. *ducreyi* in the environment is a contributing factor to the spread of the organism.

## Author summary

Children in rural communities of tropical countries often suffer skin ulcers that are caused by the bacteria *Haemophilus ducreyi*–causative agent of chancroid- and *Treponema pallidum* subsp. *pertenue* -causative agent of yaws-. The currently recommended strategy for yaws eradication is one round of mass drug administration (MDA) with azithromycin. We attempted to find reasons for the limited impact of yaws MDA on the prevalence of *H*. *ducreyi* leg ulcers by examining potential sources of infection in healthy carriers, flies, and bed linen. *H*. *ducreyi* DNA was found in skin swabs from 20% of asymptomatic children, in 9/10 flies, and 3/6 bed sheets from the houses of children with ulcers. While *H*. *ducreyi* DNA has been detected in the genital tract of asymptomatic women without genital ulcers, this is the first report of such detection on the skin of asymptomatic individuals. Importantly, skin cultures obtained from two asymptomatic children yielded viable *H*. *ducreyi*, confirming colonization and a potential reservoir of infection. If confirmed to contain viable bacteria, flies and fomites may also contribute to the continued presence of this infection after mass treatment with azithromycin. Our findings provide evidence that persistence of *H*. *ducreyi* ulcers after antibiotic MDA is due to the ubiquity of the organism in the environment. Improved hygiene and additional strategies such as repeated rounds of MDA could be able to control such a reservoir.

## Introduction

During recent yaws eradication studies, *Haemophilus ducreyi* was shown to be a major cause of chronic cutaneous ulcers in rural tropical-regions in the South Pacific islands and equatorial Africa [1–5]. Studies from yaws-endemic villages in Papua New Guinea (PNG), Vanuatu, and Ghana reported that 27% to 60% of all skin ulcers were caused by *H*. *ducreyi*, while 15% to 34% had detectable *Treponema pallidum* subsp. *pertenue* [1, 3–5]. Mixed infections with both pathogens were seen in 3% to 13% of skin ulcers. Unlike yaws, *H*. *ducreyi* infection appears to be restricted to the skin and does not result in inflammatory lesions of the bones [6]. The infectivity of *H*. *ducreyi* is high [7], and reinfections following clinical and microbiological cure of *H*. *ducreyi* genital ulcers are common [8].

A single oral dose of 30 mg/kg azithromycin is highly effective for treatment of yaws [9], while 15 mg/kg generally provides effective treatment and prophylaxis against *H*. *ducreyi* genital ulcer strains [10]. Cutaneous strains of *H*. *ducreyi* are also susceptible to macrolides in vitro [11]. In the context of yaws eradication, the use of mass drug administration (MDA) with oral azithromycin might be expected to be effective for both yaws and *H*. *ducreyi*. However, in rural villages of Lihir Island, MDA drastically decreased the proportion of ulcers containing *T*. *p*. *pertenue* DNA, but had limited impact on *H*. *ducreyi* [12].

Both infections are thought to be exclusively transmitted through non-intact skin contact with infectious lesions. Nevertheless, the persistence of *H*. *ducreyi* skin ulcers after mass azithromycin treatment [12,13] raises the possibility that the bacteria may exist in a natural reservoir. If *H*. *ducreyi* adheres and survives on the healthy skin of asymptomatic carriers, this could enhance its persistence in the community and its transmission, because skin-colonizing bacteria might escape systemic azithromycin treatment and could infect the skin after a minor abrasion. Asymptomatic colonization of exposed skin may serve as a reservoir for transmission to family members, classmates, or playmates. Asymptomatic carriage of *H*. *ducreyi* was demonstrated by PCR in the genital mucosa of about 2% of sex workers in The Gambia [14], but no studies address whether either *H*. *ducreyi* or *T*. *p*. *pertenue* potentially colonize non-genital skin.

Another source of re-infection after MDA could be fomites, such as household linens, or insects, such as flies. Linens harboring bacteria could facilitate colonization of skin or allow the bacteria to gain access to new hosts with wounds or abrasions. Indirect transmission of yaws by non-biting flies has been suggested in the older literature on the basis that *Musca* spp. and *Hippelates* spp. flies fed on scrapings from yaws lesions produced infection in experimental animals [15–17]. Infected flies were shown to have motile spirochetes (not formally shown to be *T*. *p*. *pertenue*) in their esophageal diverticula that could be regurgitated and deposited on the skin or into wounds [15,18]. Nevertheless, there is no clear evidence of *H*. *ducreyi* or yaws transmission by insects or fomites.

The demonstration of bacterial colonization or carriage usually requires a positive culture as an indication of presence and multiplication of microorganisms. However, culture of *H*. *ducreyi* is expensive and technically difficult and *T*. *p*. *pertenue* cannot be grown in vitro. Highly sensitive PCR methods cannot determine whether the source of DNA is viable organisms, but identification of bacterial DNA on the skin or a body surface is useful for exploratory analyses and helps to formulate hypotheses that could lead to new experimental studies.

The primary objective of this study was to examine the skin of asymptomatic children for the presence of *H*. *ducreyi* and *T*. *p*. *pertenue* DNA. Secondary objectives were to demonstrate the presence of viable *H*. *ducreyi* in cultures from a subset of these children, to examine whether contacts of an ulcer case are more likely to carry bacterial DNA than persons from households without ulcer disease, and to identify potential environmental sources, such as linens and flies, for the presence of *H*. *ducreyi* and *T*. *p*. *pertenue* DNA.

## Methods

### Study setting and participants

During October 2014 and May 2015 we performed a cross sectional study examining the skin of asymptomatic children and environmental sources for the presence of *H*. *ducreyi* and *T*. *p*. *pertenue* DNA in villages of Lihir Island, PNG. All villages in Lihir Island had received mass azithromycin treatment for yaws elimination in May 2013 followed by active surveillance and treatment of ulcer cases and their contacts at 6-month intervals.

Children with skin ulcers were identified during active case surveillance during the yaws elimination campaign as previously reported [12], and these were designated as “index cases”. Index cases with leg ulcers or their parents identified household relatives. We enrolled a convenience sample of asymptomatic children and young adults without ulcers from households of index cases. We also enrolled a convenience sample of asymptomatic subjects from households without cases of leg ulcers in randomly selected villages. Villages were selected with probability proportional to size sampling.

### Ethics statement

All participants, or their parents or guardians, provided written informed consent to be enrolled in this study. The protocol was approved by the National Medical Research Advisory Committee of the PNG National Department of Health (MRAC no. 17.01).

### Procedures

To assess participants for the presence of bacterial DNA, we rubbed a sterile unmoistened dacron swab on intact skin of the anterior aspect of the lower legs over a 5- by 5-cm area. Swab specimens were placed into tubes containing 1 mL of lysis buffer (10mM Tris-HCl, 0.1M EDTA, and 0.5% SDS) to stabilize DNA prior to shipment for PCR testing. Cultures were attempted on a subset of swab specimens. A structured questionnaire was administered to all asymptomatic participants to collect household-level sociodemographic information and to assess health-related, hygiene-related, and other household-level hypothesized risk factors for intra-household transmission of skin pathogens.

To assess environmental sources, we collected flies from verandas and surrounding areas immediately outside the houses of patients with cutaneous ulcers, and we swabbed the surface of their bed linens to cover 5 separate areas of 10 by 10 cm. We considered fomite (bed linens) transmission to family members, because of the standard practice of bed sharing in PNG. Flies and swabs from the linens were placed in tubes containing Tris-EDTA-SDS buffer and frozen for transportation to the laboratory.

### Laboratory assessment

The skin swab samples were sent to the University of Washington (Seattle, WA, USA) for PCR testing to detect *T*. *p*. *pertenue* and *H*. *ducreyi* DNA. As described [1], *T*. *pallidum* DNA was assayed by standard PCR for TP0548 and by TaqMan real time PCR for T47 (TP0574). The pertenue subspecies was confirmed by TprL PCR amplicon size. *H*. *ducreyi* DNA was assayed by standard PCR and by TaqMan PCR. All samples were tested by PCR for human beta-globin DNA, as a control for sample adequacy and DNA integrity.

We cultured a subset of swabs that were collected from ulcers of index cases and intact skin of asymptomatic participants for *H*. *ducreyi* on C-HgCh (Mueller Hinton agar with 5% chocolatized hemoglobin + 3 mg/ml vancomycin) culture plates in the field; these were transported in a candle jar to the laboratory within 4 hours and transferred to an incubator with BD GasPak system and incubated at 33°C for 48 h. If cultures grew organisms presumptively identified as *H*. *ducreyi* (small yellow-gray colonies whose gram stain showed small gram negative rods [6]), we transferred the plate grown colonies to a tube containing Assay Assure medium for transport, DNA extraction, and definitive species identification by PCR [19].

The flies and the Tris-EDTA-SDS buffer used for transport of flies were tested separately. Organisms passively adhering to the outside of the fly would likely be reflected in the DNA extracted from the buffer, while regurgitating pathogens would be inside the gut. Fly DNA was extracted using Gen-ial All-Tissue DNA Extraction kit. Briefly, the flies were completely disrupted in Lysis Buffer #1 using a pestle. Proteinase K, Lysis Buffer #2, and 0.01M DTT were added to the individual tubes and incubated for 48 hours at 37°C. Following centrifugation at 16,000 g, the supernatant was treated with chloroform and the aqueous phase was washed with Lysis Buffer #3. DNA was precipitated using glycogen and isopropanol. We tested each fly and transport buffer specimen using the standard PCR and TaqMan assays mentioned above. In the TaqMan assays, DNA from each fly was tested in 21 replicates (7 from the transport buffer and 14 from the extracted fly DNA). Multiple negative control PCR reactions were run in each assay, and were uniformly negative.

### Statistical analyses

The primary outcome measure was PCR-positivity rate for *T*. *p*. *pertenue* or *H*. *ducreyi* in asymptomatic participants. We compared PCR-positivity rates of contacts of an *H*. *ducreyi* ulcer case to those of participants from a household without an *H*. *ducreyi* skin ulcer case with Fisher exact tests. We reported odds ratios with 95% CIs from univariate logistic regression to compare the living conditions of PCR positive and negative participants. We accounted for clustering by household among exposed subjects using a penalized maximum likelihood estimation method in the regression model [20]. All analyses were done with Stata version 14.0.

## Results

Household visits were completed for 21 patients who had ulcerative lesions and agreed to participate. Of 21 patients with leg ulcers, 12 (57%) had detectable *H*. *ducreyi* DNA and 1 (5%) had detectable *T*. *p*. *pertenue* DNA in their ulcers; neither pathogen was detected in 8 (38%) of the index cases. Of 7 cultures obtained from leg ulcers, 2 (29%) yielded no growth and 5 (71%) yielded small gram negative rods or coccobacilli. Of the 5 gram negative organisms, 2 were confirmed by PCR to be *H*. *ducreyi*.

**Fig 1:**
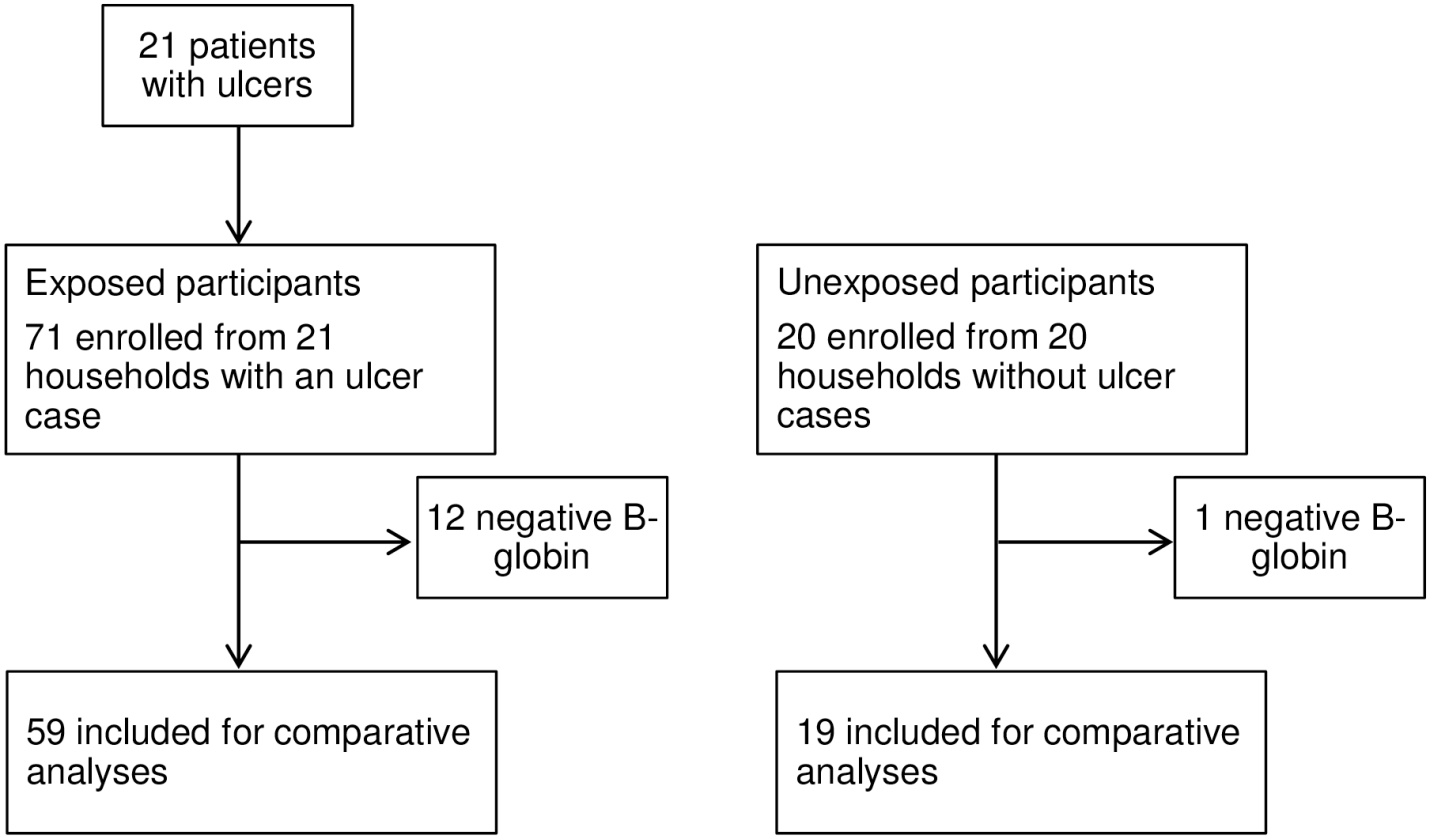
Trial profile.

We identified 71 asymptomatic subjects exposed to a skin ulcer case, and these were all enrolled (Fig 1). We also enrolled 20 asymptomatic subjects from households without an ulcer case. Of 91 asymptomatic participants tested, 12 (17%) in the group exposed to an ulcer case, and 1 (5%) in the group not exposed to an ulcer case in the household had negative beta-globin and bacterial-DNA amplification results, and were excluded from subsequent comparative analyses, so a total of 78 asymptomatic participants were evaluated.

Overall, 16 (21%, 95%CI 12–31) of 78 asymptomatic participants had *H*. *ducreyi* bacterial DNA on the skin, and 1 (1%) had *T*. *p*. *pertenue* DNA. Of 17 cultures obtained from asymptomatic contacts, 8 (47%) yielded no growth and 9 (53%) yielded small gram negative rods or coccobacilli. Six of the gram negative organisms were tested by PCR and 2 were confirmed to be *H*. *ducreyi*.

By exposure status, 8 (24%) of 34 asymptomatic participants in the group exposed to an *H*. *ducreyi* positive ulcer, and 8 (18%) of 44 participants not exposed to an *H*. *ducreyi* positive ulcer were *H*. *ducreyi* DNA positive (OR 1.38; 95%CI 0.46–4.17; p = 0.76) (Table 1, analyses by subgroups—exposed to HD negative case and not exposed to an ulcer case). The single asymptomatic participant with detectable *T*. *p*. *pertenue* DNA was identified in a household of an index case with *H*. *ducreyi* mono-infection.

**Table 1:** *H*. *ducreyi* skin PCR positivity rates.

	Group 1	Group 2	Group3	Odds ratio	Odds ratio
	Exposed to HD positive case (n = 34)	Exposed to HD negative case (n = 25)	Not exposed to ulcer case in the household (n = 19)	(95%CI) Group 1 vs. Group 2	(95%CI) Group 1 vs. Group 3
*H*. *ducreyi* DNA amplified	8 (24%)	4 (16%)	4 (21%)	1.62 (0.42–6.18)	1.15 (0.30–4.49)

HD, *H*. *ducreyi*. Data are n (%).

When we compared individual household-level socio-demographic factors, the living conditions of asymptomatic PCR-positive participants and PCR-negative participants were very similar (Table 2). Mean age (SD) of PCR-positive participants was 7.7 (3.7) years, and of PCR-negative participants was 10.3 (8.0) years. PCR-positive participants were significantly more likely to change bed linens less than once per week than participants without detectable bacterial DNA.

**Table 2:** Socioeconomic, environmental and behavioral factors that may predispose to PCR positivity with *H*. *ducreyi*.

Variable	PCR-negative (n = 61)	PCR-positive (n = 17)	OR (95%CI)	P value
Exposed to an ulcer case	46 (75%)	13 (76%)	1.06 (0.30–3.75)	0.819
Sex (male)	31 (51%)	8 (47%)	1.15 (0.41–3.28)	0.788
Age (mean, SD)^1^	10.3 (8.0)	7.7 (3.7)	0.95 (0.86–1.04)	0.280
No fixed indoor shower or bath^2^	60 (98%)	17 (100%)	0.93 (0.03–27.10)	0.967
No indoor toilet^2^	48 (79%)	17 (100%)	9.61 (0.54–169.69)	0.122
Sand or mud in the floor^2^	54 (89%)	17 (100%)	5.13 (0.27–95.88)	0.274
Traditional (temporary) housing^2^	24 (39%)	9 (53%)	1.71 (0.60–4.88)	0.319
Livestock in the house^2^	54 (89%)	17 (100%)	4.86 (0.26–89.47)	0.288
Walking bare-foot^2^	53 (87%)	17 (100%)	5.65 (0.31–103.16)	0.243
Frequency of changing bed-linen less 1 per week^3^	19 (31%)	10 (59%)	3.03 (1.03–8.87)	0.043
Frequency of bathing less 1 per day^4^	32 (52%)	9 (53%)	1.02 (0.35–2.99)	0.811
Children share towel ^2^	35 (57%)	10 (59%)	1.06 (0.36–3.10)	0.919

Univariate model adjusted for exposure to an ulcer case

^1^ Odds ratio per unit increase,

^2^ Odds ratio for yes vs. no,

^3^ Odds ratio for less 1 per week vs. more than 1 per week

^4^ Odds ratio for less 1 per day vs. more than 1 per day

Flies (n = 10) were caught outside 10 houses of patients with ulcers; 9 (90%) of 10 flies had *H*. *ducreyi* DNA and 5 (50%) had amplifiable *T*. *p*. *pertenue* DNA (Table 3). For 2 flies, *H*. *ducreyi* was detected reproducibly by standard PCR and in 21/21 replicates by real-time PCR, suggesting relatively abundant *H*. *ducrey*i on these flies. Lower amounts of *H*. *ducreyi* DNA (1–3 positive tests of 21 replicates) were found in 7 other flies. We detected *T*. *p*. *pertenue* positive results in standard and real-time PCR for 4 flies and only in real-time PCR for the 5th fly. All of the *T*. *p*. *pertenue*-positive flies also carried detectable *H*. *ducreyi* DNA. Three of the 5 flies with detectable *T*. *p*. *pertenue* DNA were collected from a community where active yaws prevalence remained high after MDA. The buffer used to transport the flies yielded amplifiable *H*. *ducreyi* DNA in 6 (60%) of 10 specimens, but none yielded amplifiable *T*. *p*. *pertenue* DNA, perhaps suggesting different location of the two bacteria in flies (i.e. *T*. *p*. *pertenue* as a regurgitating pathogen in the gut), or dilution effect related to differential bacterial load of *H*. *ducreyi* and *T*. *p*. *pertenue* carried by affected flies.

**Table 3:** Presence of *H*. *ducreyi* and *T*. *p*. *pertenue* DNA on flies.

Setting by specimen	*H*. *ducreyi* (n = 10)	*T*. *p*. *pertenue* (n = 10)
All specimens	9 (90%)	5 (50%)
Type of PCR		
Standard	5 (50%)	4 (40%)
Taqman	9 (90%)	5 (50%)
Specimen		
Whole fly	7 (70%)	5 (50%)
Lysis buffer	6 (60%)	0 (0%)

Data are n (%)

Environmental contamination was observed in 3 (50%) of 6 bed sheets sampled from 6 households with index cases; 2 sheets had detectable *H*. *ducreyi* DNA and 1 had detectable *T*. *p*. *pertenue* DNA. One of the 2 sheets with *H*. *ducreyi* and the *T*. *p*. *pertenue* sheet were from households with *H*. *ducreyi* positive index cases, while the other *H*. *ducreyi* sheet was from a household with a PCR-negative ulcer case.

## Discussion

We used PCR techniques to demonstrate that 20% of asymptomatic children living in *H*. *ducreyi*-endemic communities had detectable *H*. *ducreyi* DNA on the skin. These children had no evidence of cutaneous ulceration, and no other symptoms or signs of infection. This is the first report of asymptomatic carriage of *H*. *ducreyi* DNA on skin. Notably, people from households without an ulcer case had positivity rates that were similar to people with an *H*. *ducreyi* ulcer case in the household. Hence, a member of a household without a case should still have been exposed to an infectious case in the community, such as in school.

To detect possible *H*. *ducreyi* colonization, we chose PCR as the primary diagnostic method instead of culture because of our limited capability of performing a large number of cultures for this fastidious organism. Compared to PCR, single plate culture systems are only ~ 50% sensitive for detecting *H*. *ducreyi* in patients with chancroid [21,22], which is why PCR is the preferred diagnostic test for chancroid. However, PCR can detect nonviable bacteria or contaminating DNA; hence, a swab positive for DNA does not give definitive evidence of colonization. In our attempts to culture the organism from a subset of samples, we presumptively identified *H*. *ducreyi* in approximately half of the cultures, and two of them were positive by PCR for definitive species identification. Further studies using optimal culturing techniques are required to elucidate the true extent of the biological reservoir of *H*. *ducreyi* on the skin in this population.

Our study was significantly limited in its ability to assess asymptomatic carriage of *T*. *p*. *pertenue* because the mass azithromycin treatment conducted earlier in these communities was so effective in reducing the prevalence of yaws ulcers [12]. Since only few contacts of one yaws case were included in this study, our findings should be interpreted with caution. The viability of *T*. *p*. *pertenue* on skin surfaces or sheets is unknown. This organism is thought to be highly fragile outside of a susceptible host and would not be expected to survive on fomites.

We previously reported that approximately 2% of the total population in Lihir Island and 7% of the children aged 5–15 years had ulcers containing *H*. *ducreyi* DNA. Although *H*. *ducreyi* cutaneous ulcer strains might be exclusively transmitted through contact of wounds with infectious lesions, it seems unlikely that this mode of transmission could account for the high prevalence of infection or persistence after MDA using azithromycin. Our findings suggest that *H*. *ducreyi* survives on healthy non-genital skin where even minor trauma could initiate infection. In human volunteers, placement of up to 10^6^ colony forming units (CFU) of the genital ulcer strain 35000HP on intact skin fails to cause infection; however, as few as 1 CFU delivered by a 2 mm puncture wound is sufficient to initiate infection [7,23]. Other factors may impact the initiation of disease in carriers of *H*. *ducreyi*. For example, recent human inoculation experiments raise the possibility that the composition of the skin microbiome could influence host susceptibility to *H*. *ducreyi* skin infection and ulcer formation [24].

Our findings suggest a possible role for flies in the transmission of *H*. *ducreyi* and yaws under natural conditions. Flies have also been discussed as possible vectors of the related treponematoes, pinta and bejel [25,26] and *T*. *pallidum* DNA has been detected on flies related to treponemal infections in non-human primates [27], but direct evidence of transmission is lacking. Although we did not test whether flies carry viable bacteria, a high proportion of flies collected had detectable *H*. *ducreyi* DNA and half carried both *H*. *ducreyi* and *T*. *p*. *pertenue* DNA. Further study is necessary to unravel whether bacterial DNA in the flies reflects the ubiquity of the organism in the environment or carriage of live organisms.

The minimal effect of MDA with azithromycin on the prevalence of skin ulcers due to *H*. *ducreyi*, compared to the profound effect on the prevalence of yaws, is puzzling. *H*. *ducreyi* colonization of asymptomatic villagers, flies, and fomites could explain the continued presence of this infection after MDA. If azithromycin does not reach the outer surface of skin, it may not interrupt colonization, and these sources may perpetuate the infection in the community. Given the prolonged prophylactic effect of azithromycin against experimental *H*. *ducreyi* infection, it is plausible that repeated mass treatment can confer a prophylactic effect to the population for long enough to clear the asymptomatic reservoir [10].

New strategies to control *H*. *ducreyi* along with yaws need to be explored. Syndromic care for ulcers using azithromycin and multiple rounds of MDA could be included in future iterations of the Morges Strategy for yaws eradication. Indeed mathematical modeling has shown the value of multiple rounds of MDA to reduce *T*. *p*. *pertenue* infection [28], and this could, in parallel, reduce *H*. *ducreyi* infection and potentially skin carriage. In addition, skin hygiene and effective wound management using non-adherent dressings must be emphasized; given the potential carriage of *H*. *ducreyi* and *T*. *p*. *pertenue* by flies, covering ulcers may also help to prevent transmission.
